# Psychiatric morbidity in acromegaly: a cohort study and meta-analysis of the literature

**DOI:** 10.1007/s11102-025-01509-0

**Published:** 2025-03-13

**Authors:** Astrid Thaarup Matthesen, Christian Rosendal, Emma H. Christensen, Helga Beckmann, Frederik Østergaard Klit, Amar Nikontovic, Gustav Bizik, Peter Vestergaard, Jakob Dal

**Affiliations:** 1 Department of Endocrinology, Aalborg University Hospital, Aalborg, Denmark; 2 Steno Diabetes Center North Denmark, Aalborg University Hospital, Aalborg, Denmark; 3 Department of Psychiatry, Aalborg University Hospital, Aalborg, Denmark; 4 Department of Clinical Medicine, Aalborg University, Aalborg, Denmark

**Keywords:** Acromegaly, Psychiatric disorders, Depression, Anxiety, Pituitary adenoma

## Abstract

**Purpose:**

We aimed to evaluate the risk of psychiatric disorders through a retrospective cohort study comparing acromegaly and non-functioning pituitary adenomas (NFPAs) and a meta-analysis of existing literature.

**Methods:**

The cohort study included data from patient records analyzed using Chi^2^-, T-tests and binary regression. The meta-analysis included studies retrieved from PubMed, Embase and PsycINFO that reported risk of psychopathology in acromegaly compared to NFPA or healthy controls, using a random effects model.

**Results:**

The study population comprised 105 acromegaly and 211 NFPA patients, with similar sex distributions. Patients with acromegaly presented with smaller pituitary adenomas (17.9 (SD: 9.9) mm vs. 22.9 (SD: 10.6) mm, p < 0.001), more frequent pituitary surgery (89.1 vs. 60.2%, p < 0.001) and hormone replacement therapy (25.7 vs. 16.1%, p = 0.042). Acromegaly patients had higher risk of depression (RR: 1.9, CI95% [1.2–3.2], p = 0.009), and increased need of admissions to the psychiatric ward (5.7 vs. 0.5%, p = 0.006). The relative risk of anxiety was 1.4 (CI95% [0.5–4.4], p = 0.53). Daily opioid use was higher in acromegaly patients with psychiatric morbidity which was associated with a diagnosis of arthropathy (p = 0.009). From the meta-analysis (8 studies, 1387 patients) an increased risk of depression (RR:1.8, CI95% [1.3–2.5]) and anxiety (RR:1.9, CI95% [1.1–3.2]) was observed in acromegaly compared to NFPAs.

**Conclusion:**

This study reveals a higher risk of psychiatric disorders in acromegaly, particularly depression and anxiety. Consequently, a need for increased psychiatric awareness in acromegaly is warranted.

## Introduction

Acromegaly is caused by excessive secretion of growth hormone (GH), most often due to a GH-secreting pituitary adenoma, resulting in hypersecretion of insulin-like growth factor-1 (IGF-1) [1]. It is considered a rare disease with an earlier reported incidence rate and a prevalence of 3.8 and 85 cases per million, respectively [2]. However, recent studies report an increasing prevalence to figures of 120 cases per million [3, 4]. Disease onset is insidious, not uncommonly with a diagnostic delay of 5–10 years [2]. Excessive GH and IGF-1 cause enlargement of feet and hands, coarsening of facial features and complications such as sleep apnea, cerebrovascular disease, heart failure, hypertension, diabetes mellitus and severe arthropathy [1, 2]. Though acromegaly is still associated with increased morbidity, mortality rates have been decreasing in recent years, possibly due to improved treatment [5].

Despite improvements in treatment [5], the quality of life (QoL) in acromegaly patients remains impaired [6–8], even after long-term remission [9, 10]. When evaluating QoL, especially self-perceived appearance and physical functioning are affected [10, 11]. Impaired QoL is more frequent in females and associated with factors such as musculoskeletal pain and active acromegaly [11, 12]. In patients with prior active acromegaly, subsequent GH-deficiency has also been linked to reduced QoL [13]. Lastly, QoL is impaired by psychological symptoms associated with acromegaly, including symptoms of depression and anxiety, which may persist despite biochemical disease control [14, 15].

Generally, QoL-impairment and psychopathology are more common in patients with pituitary adenomas, including acromegaly, Cushing’s disease, prolactinomas and NFPAs [16]. Similarly, living with a chronic disease or chronic pain increases the risk of mental health problems, such as depression and anxiety [17, 18]. However, when compared to patients with chronic diseases, Sievers et al. still reported an increased risk of affective disorders in acromegaly patients [19]. Supporting these findings, a large nationwide registry-based study by Moon et al. revealed a higher risk of depression in acromegaly patients compared to healthy controls [20].

In a recent systematic review, Silvestro et al. suggested a possible association between acromegaly and depression and anxiety [21]. Yet, most of the included publications were cross-sectional studies with relatively small sample sizes and somewhat inconsistent results. Furthermore, these studies primarily relied on symptom rating scales for the assessment of depressive or anxiety-related symptoms rather than classification systems used for the diagnosing of depressive or anxiety disorders. Lastly, the included studies often used healthy subjects as controls rather than more comparable subjects, e.g. patients with NFPAs. Therefore, the present study aims to investigate the risk of psychiatric disorders, both in an unselected cohort of acromegaly patients compared to patients with NFPAs and a meta-analysis of existing literature on the topic.

## Methods

### Study population

The study population consisted of all prevalent cases of acromegaly diagnosed at the department of endocrinology in the North Denmark Region between 1977 and 2021 [5] and as a comparison cohort, all prevalent and incident cases of NFPAs followed at the department of neurosurgery Aalborg University hospital 2019–2024 were included. Data was extracted from patient medical records and included the following clinical variables: Sex, age at diagnosis, pituitary adenoma size, pituitary surgery, radiotherapy, treatment of pituitary hormone deficiency and psychiatric condition. For patients diagnosed with psychiatric disorders, additional data was collected on the type of any psychotropic drugs (antidepressive, antipsychotic, anxiolytic or other drugs), analgesics (opioids), admissions to a psychiatric ward and alcohol abuse. Patients diagnosed with a psychiatric disorder more than 10 years prior to the acromegaly or NFPA diagnosis were excluded; this cut-off was chosen based on the mean diagnostic delay of acromegaly [22] to ensure inclusion of any psychiatric disorders occurring while patients were in a state of unrecognized GH excess. For acromegaly patients, further disease-specific data was collected: Medical treatment, IGF-1-levels at diagnosis and follow-up and comorbidities (heart disease, diabetes mellitus, osteoarthritis/arthralgia, osteoporosis, sleep apnea, hypertension, carpal tunnel syndrome/polyneuropathy and cancer). Data collection was approved by local authorities (Case no. 2021-004763).

### Definition of psychiatric disorders

Psychiatric disorders were divided based on the following diagnoses: “(unipolar) depressive disorder” (referred to as “depression” in the following text for conciseness), “anxiety disorder”, “bipolar disorder”, “eating disorder”, “psychosis”, “attention deficit hyperactivity disorder (ADHD)” and “post-traumatic stress disorder (PTSD)”. A patient was classified as having one of the diagnoses above if at least one of the following criteria were met: consistent description of the diagnosis in the patients’ chart and/or prescription of a relevant psychotropic drug with a relevant therapeutic indication, noted in the patients’ chart. The last inclusion criteria ensured inclusion of as many patients as possible treated in a primary care setting. Psychoses with an organic etiology (e.g. steroids, Alzheimer’s dementia) were excluded.

### Statistical analysis

QQ-plots were used to determine whether continuous variables were normally distributed. If not, logarithmic transformation was applied. Normally distributed data was presented as mean and standard deviation (SD), and non-normally distributed data as median and interquartile range [IQR]. T-tests or rank sum tests and Chi^2^-tests or Fisher’s exact tests were used to compare continuous and binary variables between groups, respectively. Binary regressions were used to calculate risk ratios (RRs) of depression or anxiety disorder and adjusted for factors as age at pituitary disease, pituitary adenoma size, pituitary surgery, and hypopituitarism. Subgroup analyses were performed excluding NFPA patients that did not receive surgery. P-values below 0.05 were considered statistically significant. Statistical analysis was performed using Stata Version 18.

### Meta-analysis

To identify published studies concerning the risk of psychiatric morbidity in patients with acromegaly, the databases PubMed, Embase and PsycINFO were searched in September 2024. Three separate search strings for each database, containing both index search terms and free text search, consisted of two cornerstones: Acromegaly and mental disorder. The index term “acromegaly*” was combined with “mental disorder*” or “mental disease*” or “behavior disorder*” or “psychiatric diagno*” or “severe mental disorder*” or “mental health*” or “psychiatric symptom*” or “psychopatholog*”.

Studies enabling the calculation of RRs for psychiatric morbidity in acromegaly patients compared to a relevant control group (healthy subjects or NFPA patients) were included. Based on the included studies, RRs were calculated, and a meta-analysis performed. Due to non-normality of the results, RRs and their 95% confidence intervals (CIs) were log-transformed. Due to between-study heterogeneity, a random effects model (restricted maximum likelihood) was applied.

## Results

### Clinical characteristics

The study included 105 acromegaly patients (Table 1). The mean IGF-1-level at diagnosis was 2.3 (1.8) times upper limit of normal (xULN). At follow-up, 91% of the patients with acromegaly were biochemically controlled [3].

**Table 1:** Patient characteristics of acromegaly and NFPA patients (*n* = 316)

		Acromegaly patients (*n* = 105)	NFPA patients (*n* = 211)	*P-value*
Age at pituitary diagnosis (years, mean (SD))		48.8 (14.9)	57.2 (16.6)	< 0.001*
Sex (female (%, n))		54 (57)	45 (94)	0.10
Pituitary adenoma size (mm, mean (SD))		17.9 (9.9)	22.9 (10.6)	< 0.001*
Any pituitary surgery (%, n)		85.7 (90)	60.2 (127)	< 0.001*
Number of surgeries (%, n)	1	78.9 (71)	79.5 (101)	0.075
	2	21.1 (19)	15.7 (20)	
	≥ 3	0.0 (0)	4.7 (6)	
Radiation therapy (%, n)		11.4 (12)	3.3 (7)	0.003*
Medical treatment for acromegaly (%, n)		40.0 (42)		
No. of comorbidities (median) [IQR]		2 [2, 3]		
IGF-1 at diagnosis (xULN, mean(SD))		2.3 (1.8)		
IGF-1 at follow-up (xULN, mean (SD))		0.9 (1.8)		
Any hormone replacement therapy (%, n)		25.7 (27)	16.1 (34)	0.042*
Number of replaced axes (%, n)	1	48.2 (13)	61.8 (21)	0.59
	2	29.6 (8)	17.6 (6)	
	3	14.8 (4)	17.6 (6)	
	4	7.4 (2)	2.9 (1)	
Psychiatric diagnoses any (%, n)		27.6 (29)	14.7 (31)	0.006*

Statistically significant results are marked with *

*IGF-1* Insulin-like Growth Factor-1, *xULN* times Upper Limit of Normal, *SD* standard deviation, *IQR* Interquartile range, *NFPA* non-functioning pituitary adenoma

In the group of acromegaly patients, no significant differences were observed between patients with or without psychiatric diagnoses as regards to age at pituitary diagnosis, sex, pituitary adenoma size, treatment (both surgical, medical and radiation), number of comorbidities, IGF-l-level at diagnosis and at follow-up or pituitary insufficiency (data not shown). The same accounted for all comorbidity, except for hypertension that was more prevalent in the group of acromegaly with a psychiatric diagnosis (p < 0.05) (data not shown).

Acromegaly patients were diagnosed at a younger age (48.8 ± 14.9 years) compared to NFPA patients (57.2 ± 16.6 years, p < 0.001), with similar sex distribution (Table 1). Pituitary adenomas were smaller in patients with acromegaly (17.9 ± 9.9 mm) than in patients with NFPAs (22.9 ± 10.6 mm, p < 0.001). More acromegaly patients were operated compared to NFPA patients (85.7 vs. 60.2%, p < 0.001). Among operated patients, the number of surgeries did not differ between the two groups (p = 0.075). More acromegaly patients received replacement therapy for hypopituitarism compared to NFPA patients (25.7 vs. 16.1%, p = 0.042). Among patients treated for hypopituitarism, the number of replaced hormone axes did not differ between groups (p = 0.59). The risk of psychiatric diagnoses was significantly higher in acromegaly patients (27.6%) compared to NFPA patients (14.7%, p = 0.006).

### Clinical characteristics of patients with psychiatric diagnoses

In pituitary patients with a psychiatric diagnosis, a larger proportion of acromegaly patients were operated compared to NFPA patients (p = 0.02), while the number of surgeries in operated patients was comparable (p = 0.33) (Table 2). The groups were comparable regarding age at pituitary diagnosis, sex, pituitary adenoma size and pituitary insufficiency.

**Table 2:** Patient characteristics of the acromegaly and NFPA patients with psychiatric diagnoses (*n* = 60)

		Acromegaly patients (*n* = 29)	NFPA patients (*n* = 31)	*P*
Age at pituitary disease (years, mean (SD))		47.2 (15.6)	52.4 (17.2)	0.23
Sex (female (%, n))		62 (18)	45 (14)	0.19
Pituitary adenoma size (mm, mean (SD))		18.2 (10.5)	22.8 (12.3)	0.16
IGF-1 at diagnosis (xULN, mean (SD))		2.4 (2.1)		
IGF-1 at follow-up (xULN, mean (SD))		0.9 (1.9)		
Any pituitary surgery (%, n)		82.8 (24)	58.1 (18)	0.02*
Number of surgeries (%, n)	1	83.3 (20)	77.8 (14)	0.33
	2	16.7 (4)	11.1 (2)	
	≥ 3	0.0 (0)	11.1 (2)	
Any hormone replacement therapy (%, n)		24.1 (7)	16.1 (5)	0.44
Number of replaced axes (%, n)	1	57.1 (4)	60.0 (3)	0.74
	2	42.9 (3)	20.0 (1)	
	3	0.0 (0)	20.0 (1)	
Age at psychiatric diagnosis (years, mean (SD))		56.5 (17.3)	52.6 (18.9)	0.41
Time from pituitary disease to psychiatric diagnosis (years, median [IQR])		6.0 [0.9–16.5]	− 0.2 [− 3.5 to 3.4]	0.002*
Depression (%, n)*		23.8 (25)	12.3 (26)	0.009*
Anxiety disorder (%, n)*		4.8 (5)	3.3 (7)	0.53
Admissions to a psychiatric ward (%, n)*		5.7 (6)	0.5 (1)	0.006*
Daily opioid use (%, n)		27.6 (8)	0.0 (0)	NA

* % of the whole cohort (*n* = 105 and *n* = 211 for acromegaly and NFPA patients, respectively)

*IGF-1* Insulin-like Growth Factor-1, × *ULN* Times Upper limit of normal, *SD* standard deviation, *IQR* interquartile range, *NFPA* non-functioning pituitary adenoma

The age at psychiatric diagnosis did not differ between groups (p = 0.41). However, patients with acromegaly were diagnosed 6.0 [0.9, 16.5] years after their pituitary diagnosis, while patients with NFPAs were diagnosed 0.2 [− 3.5, 3.4] years prior to their pituitary diagnosis (p = 0.002).

Depression predominated in both groups, but the risk was significantly higher in acromegaly patients compared to patients with NFPAs (23.8 vs. 12.3%, p = 0.009). Anxiety disorder was the second-most frequent diagnosis (4.8% in acromegaly patients vs. 3.3% in NFPA patients, p = 0.53). Bipolar disorder was less frequent but similar in both acromegaly and NFPA patients (1.0 vs. 0.5%, p = 0.55). In acromegaly patients, psychoses (1.0%) and eating disorders (1.9%) were observed, the latter comprising one patient with an unspecified eating disorder, and one with overeating associated with other psychological disturbances. In relation to the acromegaly diagnosis, the patients were diagnosed approximately 7 years before and 4 years after, respectively. Diagnoses of ADHD (*n* = 2) and PTSD (*n* = 1) were only present in NFPA group.

All patients with psychiatric diagnoses received psychotropic medications, except for one NFPA-patient and two acromegaly patients. A larger proportion of acromegaly patients were treated with antidepressant drugs compared to NFPA patients (24.8 vs. 13.7%, p = 0.02). The use of antipsychotic (4.8 vs. 1.4%, p = 0.12) or anxiolytic drugs (5.7 vs. 1.9%, p = 0.07) did not differ significantly between groups. Other psychotropic medications were used by 1.9% of acromegaly patients and 2.4% of NFPA patients, including mood-stabilizers (lithium, lamotrigine and valproate) and methylphenidate.

Significantly more acromegaly patients were admitted to a psychiatric ward compared to NFPA patients (5.7 vs. 0.5%, p = 0.006). Among acromegaly patients with psychiatric diagnoses, a daily use of opioids was noted in 27.6% with a median morphine equivalent dose of 25 [20–45] mg. Opioid use was associated with an increased risk of arthropathy in the acromegaly cohort (p = 0.009) (data not shown).

### Binary regressions and the risks of depression and anxiety disorders

Using a binary regression model, acromegaly patients had a significantly higher risk of depression compared to NFPA patients, with an unadjusted RR of 1.93 (95% CI [1.18, 3.17], p = 0.009). When adjusting for factors including age at pituitary disease, pituitary adenoma size, pituitary surgery and hypopituitarism, the increased risk retained statistical significance (RR 1.86, 95% CI [1.02, 3.39], p = 0.04). In a subgroup analysis excluding NFPA patients without pituitary surgery, acromegaly patients had an increased risk of depression compared to NFPA patients (RR 1.89, 95% CI [1.07, 3.35], p = 0.03).

The risk of anxiety disorder in acromegaly compared to NFPA was 1.44 (95% CI [0.47, 4.41], p = 0.53) for the unadjusted model. In subgroup analysis excluding all NFPA patients without pituitary surgery, the relative risk was 1.01 (95% CI [0.32, 3.21, p = 0.99).

### Meta-analysis

**Fig. 1:**
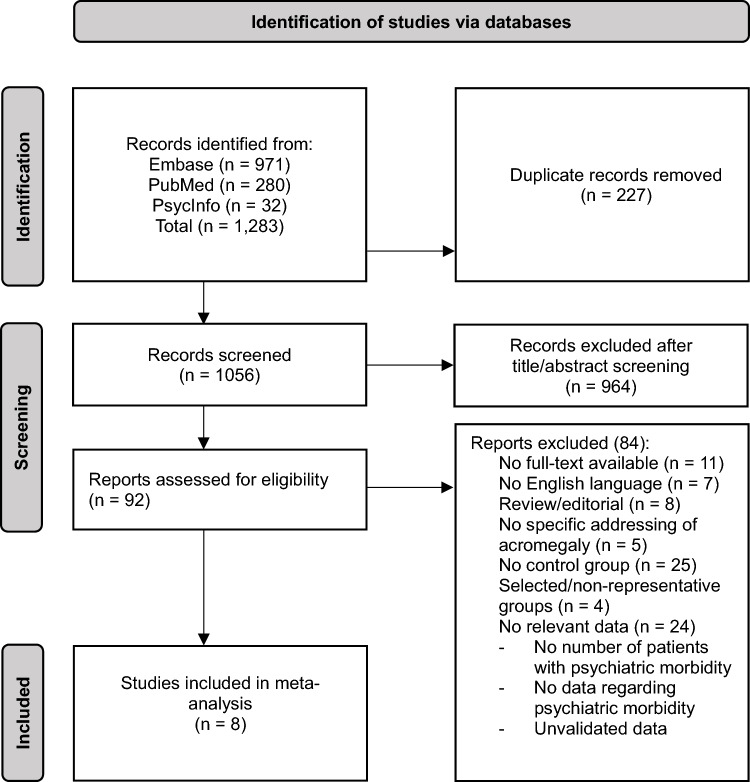
PRISMA flowchart

A total of 1283 publications were identified, of which 227 duplicates were removed. Of the remaining publications, 964 were excluded based on title or abstract. Following a detailed evaluation of the remaining 92 publications, 8 studies with a total of 1387 patients were deemed eligible for inclusion based on presentation of data regarding the prevalence of psychopathology or psychiatric diagnoses among acromegaly patients, compared to a control group of either healthy subjects or NFPA patients (Fig. 1, Table 3).

**Table 3:** Characteristics of the studies included in the meta-analysis (n = 8)

Authors	Year	Study design	Country	Population	Number of patients with psychiatric morbidity
Algahtany et al. [23]	2021	Cohort CES-D scale	Canada	15 acromegaly 20 NFPA	Depression Acromegaly: 3 (20%) NFPA: 4 (20%)
Anagnostis et al. [24]	2013	Cross-sectional POMS scale	Greece	40 acromegaly 80 healthy	Depression Acromegaly: 5 (12.5%) Healthy: 3 (3.9%)
Conaglen et al. [25]	2015	Cross-sectional HADS scale	New Zealand	32 acromegaly 29 NFPA 21 healthy	Depression/anxiety Acromegaly: 12 (37.5%) NFPA: 7 (24.1%) Healthy: 3 (14.3%)
Durcan et al. [26]	2021	Cross-sectional STAI-S scale	Turkey	217 acromegaly 137 healthy	Anxiety Acromegaly: 105 (48.4%) Healthy: 68 (49.6%)
Leon-Carrion et al. [27]	2010	Cross-sectional BDI-II scale	Spain	16 acromegaly 16 healthy	Depression Acromegaly: 10 (62.5%) Healthy: 2 (15.4%)
Sievers et al. [19]	2009	Cross-sectional DIA-X/M-CIDI (diagnosis)	Germany	81 acromegaly 430 healthy	Depression Acromegaly: 23 (28.4%) Healthy: 50 (11.6%) Anxiety Acromegaly: 0 Healthy: 8 (1.9%)
Tiemensma et al. [28]	2010	Cross-sectional HADS scale	Netherland	68 acromegaly 68 healthy	Depression/anxiety Acromegaly: 13 (19%) Healthy: 7 (10%)
Zhang et al. [29]	2020	Cohort Self-rating anxiety scale, self-rating depression scale scale	China	39 acromegaly 78 NFPA	Depression Acromegaly: 19 (48.7%) NFPA: 19 (24.4%) Anxiety Acromegaly: 8 (20.5%) NFPA: 5 (6.4%)

*CES-D* Center for Epidemiological Studies Depression, *POMS* profile of mood states, *HADS* hospital anxiety and depression scale, *STAI* state-trait anxiety inventory—state anxiety subscale, *BDI-II* beck depression inventory-II, *DIA-X/M-CIDI* Diagnostisches Expertensystem für psychische Störungen/Munich-Composite International Diagnostic Interview, *NFPA* non-functioning pituitary adenoma

**Fig. 2:**
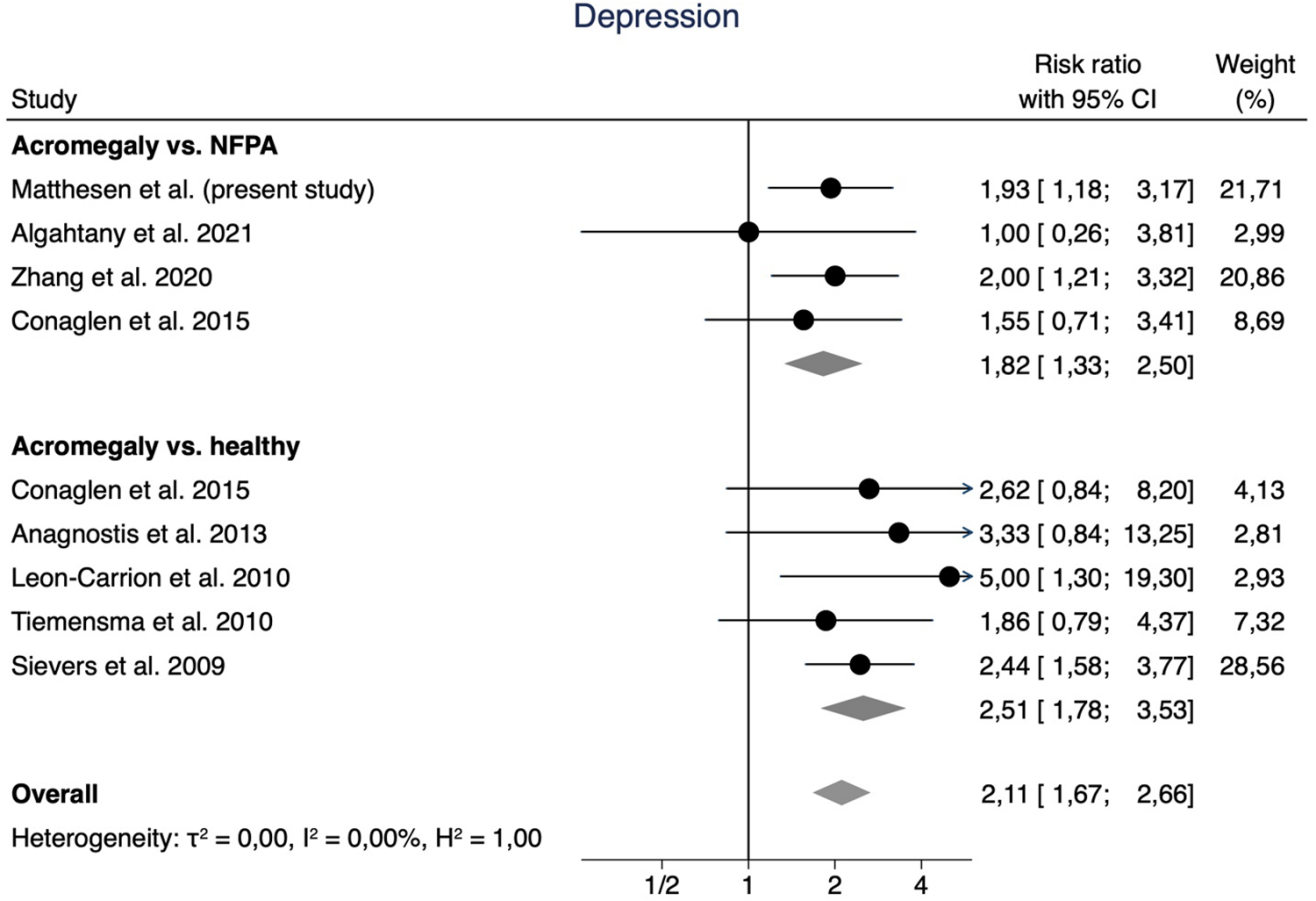
Forest plot of the risk of depression in acromegaly patients compared to NFPA patients and healthy controls. A*bbreviations*: RR: Risk Ratio, CI: Confidence Interval

**Fig. 3:**
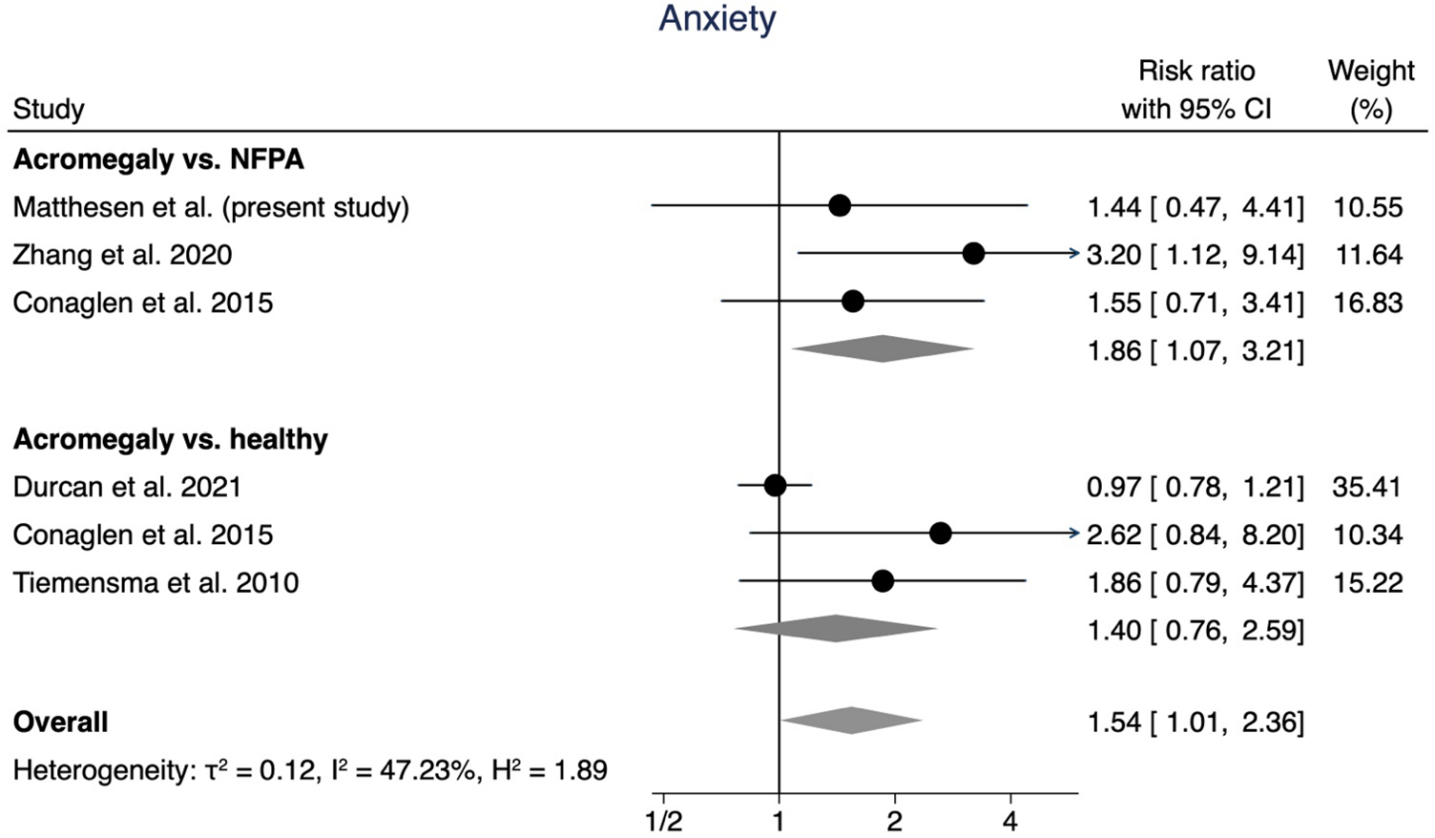
Forest plot of the risk of anxiety in acromegaly patients compared to NFPA patients and healthy controls. *Abbreviations*: RR: Risk Ratio, CI: Confidence Interval

Based on the available data, the meta-analysis was divided into two categories: The risk of depression (Fig. 2) or anxiety (Fig. 3). These categories were further divided by comparison cohorts: “acromegaly vs. NFPA” and “acromegaly vs. healthy”.

The meta-analysis revealed a higher pooled risk of depression in acromegaly patients (RR 2.11, 95% CI [1.67, 2.66]), with low heterogeneity (*I*^2^ = 0.0%). When comparing acromegaly patients to NFPA patients, the risk remained increased (RR 1.82, 95% CI [1.33, 2.50]). Also, the risk was increased 2.51 times compared to healthy controls (95% CI [1.78, 3.53]).

Likewise, the meta-analysis showed an increased pooled risk of anxiety in acromegaly patients (RR 1.54, 95% CI [1.01, 2.36]), with low-moderate heterogeneity (*I*^2^ = 29.55%). When comparing to NFPA patients, the risk of anxiety in acromegaly patients was 86% higher (RR 1.86, 95% CI [1.07, 3.21]). Compared to healthy controls, the RR was increased at 1.40, but did not reach statistical significance (95% CI [0.76, 2.59]).

## Discussion

The main finding of this study was the markedly increased risk of psychiatric disorders, particularly depression, in patients with acromegaly compared to patients with NFPAs. The risk was increased almost two-fold, corresponding to nearly a quarter of acromegaly patients being diagnosed with depression. Additionally, significantly more acromegaly patients than patients with NFPAs were admitted to the psychiatric ward. This, along with the use of psychotropics in all but two cases, further underscores the severity of psychiatric morbidity in acromegaly. These findings were supported by the meta-analysis, which revealed a significantly higher risk of depression in acromegaly patients, both compared to healthy controls and patients with NFPAs, a patient group known to exhibit impaired QoL and more depressive symptoms than healthy controls [28, 30]. Similarly, the meta-analysis revealed a significantly higher risk of anxiety in acromegaly patients compared to NFPA patients. However, no such difference was found in our cohort study, most likely due to insufficient statistical power. Held together, the cohort study and meta-analysis strongly suggest a clinically relevant problem of depression and anxiety among acromegaly patients.

Previous literature has demonstrated a worsening of psychological symptoms in patients with long-term cure of GH-excess in acromegaly patients compared to NFPA patients [9, 28], suggesting that acromegaly patients, even several years from diagnosis of pituitary disease and regardless of biochemical control, should be considered at risk of mental health problems, including depression. Acromegaly disease activity, as judged by IGF-1-levels, did not differ between the subgroup of acromegaly patients with or without psychiatric diagnoses, neither at time of acromegaly diagnosis, nor after 3–5 years of follow-up. Moreover, the median time from pituitary disease to psychiatric diagnosis was significantly longer in acromegaly patients (6 years) than in NFPA patients (− 0.2 years). By then, approximately 90% of this cohort had achieved biochemical control [3], suggesting that the effects of previous GH excess, rather than active acromegaly, may contribute to the observed psychiatric morbidity. The impact of GH and IGF-1 on QoL, depression and anxiety scores has been widely debated in former studies with inconsistent findings [31]. It has been hypothesized, that the transition from prolonged GH-excess to relative GH-deficiency after treatment may affect mental health [13], a form of withdrawal phenomenon also seen in androgen abuse [32] and after surgical treatment of patients with Cushing’s disease [33]. The delayed appearance of psychiatric morbidity in acromegaly may also be influenced by the effects of acromegaly complications compounded by the effects of natural ageing. Elderly acromegaly patients have been found to exhibit worse cognitive function, functional mobility and impaired activities of daily living, and more joint complaints, despite less aggressive disease [34].

Factors such as musculoskeletal pain could potentially contribute to psychiatric morbidity. Persistent joint pain after long-term remission has been linked to impaired QoL [12, 35]. However, we did not observe differences in the risk of comorbidities (except hypertension) between acromegaly patients with and without psychiatric diagnoses. Nevertheless, the severity of arthropathy may differ as daily opioid use was observed in 28% of acromegaly patients with psychiatric morbidity and was associated with the risk of arthropathy. This figure is slightly higher than observed in our national cohort of acromegaly patients, where 25% had filled a prescription for opioids to treat musculoskeletal pain (unpublished data). In contrast, none of the patients with NFPA and a psychiatric diagnosis had a daily use of opioids. The clinical characteristics of pain in pituitary adenomas have been investigated [36], showing that pain and pain-related disability correlate significantly with depression and impaired QoL. Recent reviews have addressed an overlap between chronic pain and depression, with common neuroplasticity changes [37, 38] and a reciprocal relationship between pain and depression, with pain as a strong predictor of subsequent depression severity [39]. Thus, pain should be addressed as a risk factor and possible target for intervention when diagnosing and treating depression in acromegaly patients.

Of other psychiatric diagnoses, two acromegaly patients (1.9%) were diagnosed with an eating disorder. Both patients were females diagnosed with concomitant depression and eating disorder. A previous study investigating the psychological profile of 223 acromegaly patients showed that scores for weight phobia, body image concerns, avoidance, compulsive self-monitoring and depersonalization were significantly higher in women [14]. Possibly, female acromegaly patients are more prone to eating disorders due to the concomitant physical changes, and a link between depression and body image perception has been reported [40]. These findings suggest that acromegaly patients may be at risk of developing eating disorders, owing to a combination of physical changes, altered body image and increased risk of depression.

The results of our cohort study showed no statistically significant differences in sex distribution among acromegaly patients with or without psychiatric diagnoses, nor between acromegaly and NFPA patients with psychiatric diagnoses. This contrasts with previous literature, where several studies found that female acromegaly patients exhibit significantly worse QoL, depression and anxiety scores than males [11, 21, 35, 41]. Nevertheless, we did observe a tendency toward female overrepresentation in the group of acromegaly patients with psychiatric morbidity, of which 62% were female, as opposed to 45% in NFPA patients with psychiatric morbidity.

This study has several strengths, including a large sample size, a well-defined catchment area with a high prevalence of acromegaly [3] and chart review to validate diagnoses of both acromegaly and psychiatric disorders. Moreover, the control group consisted of patients with NFPAs, thus minimizing confounding factors such as tumor-related mass-effect, pituitary surgery and other potential psychological and physical burdens of having a pituitary adenoma, thereby isolating the effects on mental health attributable to acromegaly alone. Other registry-based studies concerning acromegaly and psychiatric disorders compared healthy individuals and patients with other chronic somatic disorders [19, 20]. Lastly, we examined the risk of having a psychiatric diagnosis whereas previous studies examining the mental health among acromegaly patients mostly relied on symptom rating scales. A limitation of this study included its retrospective nature, which entailed risk of missing data, and controls were not age-and-sex-matched; this was however addressed by adjusting for these factors and other baseline differences. Finally, given the study design, we had no direct measurement of psychiatric disease severity, nor can we exclude socioeconomical and demographical variations between acromegaly and NFPA patients, although we expect comparable conditions due to data stemming from only one region. The results of the meta-analysis may be affected by heterogeneity in the included studies, as seven different self-report scales with no robustly established cut-off scores were used, as well as two different interview-based assessments. One study did not clearly define depression [24], whereas others reported data on depression and anxiety pooled together [25, 28], further complicating interpretation of the results. Thus, it is important to consider that the categories 'depression' and 'anxiety' refer therefore to a more heterogeneous patient group, although still with clinically significant psychopathology.

In conclusion, this study reveals a markedly increased risk of psychiatric morbidity in acromegaly patients, particularly depression, supported by the meta-analysis showing a higher risk of both depression and anxiety. The psychiatric burden of acromegaly is significant, with increased risk of needing psychotropic medication and in some cases admissions to the psychiatric ward. Furthermore, a possible reciprocal association between pain and depression in acromegaly patients warrants consideration of systematic screening for depression, as routine screening for depression in the general population is not recommended by the current psychiatric guidelines. Additionally, eating disorders may be an overlooked comorbidity in acromegaly. As such, a greater focus on the psychological profile of acromegaly patients, even several years after diagnosis and despite biochemical control, should be implemented in daily patient care among treating physicians to ensure early diagnosis and sufficient treatment.

## Data Availability

No datasets were generated or analysed during the current study.
